# Association between maternal distress during pregnancy and lower 5-min-Apgar score of the offspring: the Japan Environment and Children’s Study

**DOI:** 10.1265/ehpm.24-00305

**Published:** 2025-04-15

**Authors:** Gita Nirmala Sari, Satoyo Ikehara, Kanami Tanigawa, Yoko Kawanishi, Ehab S. Eshak, Tadashi Kimura, Tomotaka Sobue, Hiroyasu Iso

**Affiliations:** 1 Department of Environmental Medicine and Population Sciences, Graduate School of Medicine, Osaka University, Suita, Osaka, Japan; 2 Osaka Regional Center for Japan Environment and Children’s Study (JECS), Osaka University, Suita, Osaka, Japan; 3 Department of Midwifery, Polytechnic of Health, Ministry of Health Jakarta III, Jakarta, Indonesia; 4 Maternal & Child Health Information Center, Osaka Women’s and Children’s Hospital, Izumi, Osaka, Japan; 5 Obstetrics and Gynecology, Osaka University Graduate School of Medicine, Suita, Osaka, Japan; 6 Public Health, Faculty of Medicine, Minia University, Minia, Egypt; 7 Global Health Department, Denison University, Ohio, USA; 8 Institute for Global Health Policy Research, Bureau of International Health Cooperation, National Center for Global Health and Medicine, Shinjuku, Tokyo, Japan

**Keywords:** Maternal distress, Kessler Distress Scale, Apgar score, Birth cohort

## Abstract

**Background:**

Although the influence of maternal distress during pregnancy on newborn Apgar scores has been studied in various populations, there is limited research specifically addressing this issue among Asian women. This study of Japanese women aims to investigate the association between maternal distress during pregnancy and the risk of a low 5-min-Apgar score among newborns.

**Methods:**

We analyzed data from 87,765 mother-newborn pairs in the Japan Environment and Children’s Study. Using multivariable logistic regression, we estimated odds ratios (OR) and 95% confidence intervals (CI) for low Apgar scores (<7) at 5 minutes about maternal distress during early and mid-late pregnancy, as measured by the Kessler Psychological Distress Scale (K6). Apgar scores were obtained from newborns’ medical records.

**Results:**

A higher risk of low Apgar score in newborns at 5 minutes was found in mothers with moderate to severe distress than in those with low distress during mid-late pregnancy. The adjusted OR (95% CI) was 1.22 (1.05–1.42) for moderate distress (K6 = 5–12) and 1.42 (1.00–2.01) for severe distress compared to low distress (p for trend = 0.002). The positive association between maternal distress and the risk of low Apgar score was observed in preterm birth (<37 weeks) and low birth weight (<2,500 g) but not in term birth and normal birth weight.

**Conclusion:**

Maternal distress during mid-late pregnancy was positively associated with the risk of low Apgar score of newborns, specifically in preterm birth and low birth weight.

## Background

Pregnant women often experience psychological distress during pregnancy [1]. Prenatal distress has a direct effect on infant health and adverse outcomes through stimulating the hypothalamic-pituitary-adrenal (HPA) axis and an indirect effect through maternal illness accompanied by poor nutrition, reduced physical activity, and disordered sleep [2].

One of the major clinical outcomes for infant health is the newborn’s condition assessed by the Apgar score [3], determining the need for resuscitation and assessing the risk of neurologic disability and mortality [4]. A large multiethnic cohort study of 8,050 pregnant women in the Netherlands reported that depressive symptoms, as assessed with a score of ≥16 with the Center for Epidemiologic Studies Depression (CES-D) scale, at the second trimester of gestation was associated with an increased risk of low 5-min Apgar score (<7) with the multivariable OR of 1.74 (95% CI: 1.13–2.69) [5]. That association did not differ among Dutch, Creole, Turkish, and Moroccan. No such research has been conducted in Eastern countries, including Japan, but the research would be important because maternal distress and depressive symptoms could be influenced by social and ethical backgrounds. Therefore, this study aims to investigate the association between the mothers’ distress during pregnancy and the risk of a low 5-min Apgar score of newborns.

## Methods

### Study population and setting

The present study used the data from the Japan Environment and Children’s Study (JECS), recruiting pregnant women between January 2011 and March 2014. The research concept and protocol of JECS were reported comprehensively elsewhere [6, 7]. Briefly, JECS is funded by Japan’s Ministry of the Environment and involves the collaboration between the Programme Office (National Institute for Environmental Studies), the Medical Support Centre (National Centre for Child Health and Development), and 15 Regional Centers located across Japan.

The research data was based on the jecs-ta-20190930 data set, released in October 2019, revised in February 2020, and included 104,062 records. The information on pregnant women in the first to third trimesters was collected during antenatal check-ups using a questionnaire and included the medical records of the examination. Physicians, midwives/nurses, and/or research coordinators performed data transcription of the medical record.

Of the 104,062 records, we excluded 1,992 records of mothers with multiple pregnancies, 1,535 records of mothers whose pregnancies ended with miscarriage or stillbirth, and 7,297 records with missing and invalid newborn Apgar score data. We further excluded 4,653 records of mothers who reported a history of mental illness during pregnancy (depression and stress/anxiety illness), 117 mothers with history of taking medicine for mental health disorder (anti-psychotic drug) since before pregnancy, six records for missing data on the sex of the newborn, 676 records of newborn with a history of heart and lung congenital defect [8], six records of missing data for mother’s age, and 15 records for missing data on birth weight.

**Fig. 1:**
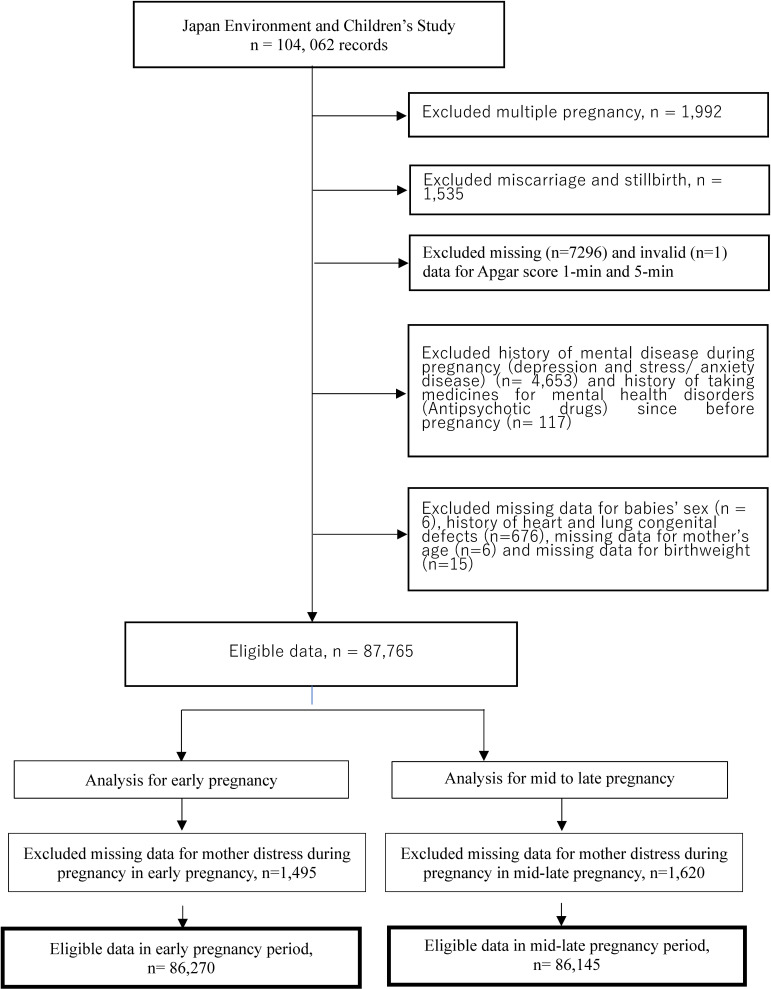
Flow chart for the calculation of respondents

The total sample size in this study consisted of 87,765 pairs of mothers and newborns. Furthermore, we excluded 1,495 and 1,620 records because of missing data on mothers’ distress in the early and mid-late pregnancy periods, respectively. Finally, we used 86,270 mothers and newborns in the early pregnancy period and 86,145 mothers and newborns in the mid-late pregnancy period as the final samples for data analyses (Fig. 1).

### Assessment of distress during pregnancy

We used the Kessler Psychological Distress Scale (K6) to assess distress during the first trimester (early pregnancy, 0–12 weeks) and between the second and third trimesters (mid-late pregnancy, 12–27 and 28–40 weeks). K6 is a psychological measurement tool used to measure distress in adults, and a Japanese version has also been adapted [9]. In this study, we categorized pregnant women into the following three groups according to the K6 score: low (<5), moderate (5–12), and severe distress (≥13).

### Assessment of outcome

The outcome in this study was the Apgar score at 5 minutes of the newborn’s life. The Apgar score is a scoring system used to assess the condition of newborns at 1 and 5 minutes after birth, which is carried out by health workers easily and quickly without the need for special equipment (includes heart rate, respiratory effort, reflex irritability, muscle tone, and color) [10] Apgar score is still used to assess the newborns’ physical condition immediately after birth to predict survival of neonates [11]. The total Apgar score (Apgar score = 10) is obtained from the sum of the scores of all the components of the Apgar score assessment; a score of ≥7 indicates that the baby is in good condition [11]. We retrieved the Apgar score data from the medical records of the newborns’ physical examination (diagnosed by a doctor). We dichotomized the 5-min Apgar score into a low (<7) and a standard Apgar score (≥7).

### Statistical analysis

The characteristics of pregnant women and newborns were summarized using descriptive statistics. Continuous variables are presented as mean values with standard deviations, while categorical variables are expressed as proportions across the three categories of maternal distress.

To assess the association between maternal distress and the risk of a low 5-min Apgar score (<7), we performed logistic regression analyses. Odds ratios (ORs) and 95% confidence intervals (CIs) were calculated to estimate the strength of the association. Separate models were constructed for maternal distress during early pregnancy (first trimester) and mid-late pregnancy (second and third trimesters). Adjustments were made for potential confounders, including mothers’ age, education (≤high school or college/university) and smoking during pregnancy (never, quit before pregnancy, quit after being pregnant, or currently smoking), parity (0 or ≥1), mode of delivery (spontaneous delivery, induction of labor, vacuum/forceps, or cesarean section), preexisting hypertension (yes or no), pregnancy-induced hypertension (yes or no), preexisting diabetes mellitus (yes or no), gestational diabetes mellitus (yes or no), hypothyroidism (yes or no), anemia medication/iron pill (yes or no) and congenital malformation (yes or no). For sensitivity analysis, we combined moderate and severe distress (K6 ≥ 5) into a single category and recalculated the ORs in comparison to the low distress group (K6 < 5). We also estimated ORs for every five-point increment in the K6 score to evaluate a dose-response relationship.

Stratified analyses were performed by gestational age at birth (<37 weeks or ≥37 weeks) and birth weight (<2500 g or ≥2500 g). Cross-product terms between maternal distress and these stratification variables were included in the logistic regression models to test for statistical interactions.

All missing observations for covariates were treated as dummy variables to maintain the sample size. The statistical analysis was conducted using SAS statistical software version 9.4 (SAS Institute Inc., Cary, NC, USA). The statistical significance was set at a two-tailed p-value of <0.05.

### Ethical statement

The JECS protocol was reviewed and approved by the Ministry of the Environment’s Institutional Review Board on Epidemiological Studies and the Ethics Committees of all participating institutions. Written informed consent was obtained from all participants.

## Results

### Participant’s characteristics

The characteristics of pregnant women and newborns with respect to maternal distress during the pregnancy period are presented in Table 1. Of the 86,270 participants in early pregnancy and 86,145 participants in mid-late pregnancy, the incidence of low 5-min Apgar scores was 1.1% (922 newborns) and 1.0% (875 newborns) of the total participants, respectively.

**Table 1:** Baseline characteristics according to psychological distress during pregnancy in early and mid-late pregnancy periods

**Parameters**	**Distress in early pregnancy period** K6* score (median)			**Distress in mid-late pregnancy period** K6* score (median)		
	
	**0–4 (1)**	**5–12 (7)**	**≥13 (14)**	**0–4 (1)**	**5–12 (7)**	**≥13 (15)**
Number at risk, n (%)	59,983 (69.5)	23,757 (27.5)	2530 (2.9)	62,401 (72.4)	21,342 (24.8)	2,402 (2.8)
Mother age at delivery, mean (SD)	31.5 (5.0)	30.6 (5.1)	29.5 (5.3)	31.5 (4.9)	30.5 (5.2)	29.2 (5.6)

*Obstetrical factors*						
Parity, n (%)						
0	22,291 (37.2)	10,191 (42.9)	1123 (44.4)	23,831 (38.2)	8767 (41.1)	994 (41.4)
≥1	36,397 (60.7)	12,994 (54.7)	1349 (53.3)	37,148 (59.5)	12,119 (56.8)	1346 (56.0)
Missing	1295 (2.2)	572 (2.4)	58 (2.3)	1422 (2.3)	456 (2.1)	62 (2.6)
Gestational age, n (%)						
≤36 weeks (preterm)	2613 (4.4)	1074 (4.5)	117 (4.6)	2521 (4.0)	1045 (4.9)	134 (5.6)
≥37 weeks (term)	57,370 (95.6)	22,683 (95.5)	2413 (95.4)	59,880 (96.0)	20,297 (95.1)	2268 (94.4)
Birthweight, n (%)						
<2500 g	4609 (7.7)	1934 (8.1)	215 (8.5)	4621 (7.4)	1807 (8.5)	226 (9.4)
≥2500 g	55,374 (92.3)	21,823 (91.9)	2315 (91.5)	57,780 (92.6)	19,535 (91.5)	2176 (90.6)
Mode of delivery						
Spontaneous delivery	34,875 (58.1)	13,609 (57.3)	1461 (57.8)	36,245 (58.1)	12,273 (57.5)	1384 (57.6)
Induction of labor	10,451 (17.4)	4297 (18.1)	443 (17.5)	11,058 (17.7)	3779 (17.7)	385 (16.0)
Vacuum/Forceps	3298 (5.5)	1498 (6.3)	149 (5.9)	3484 (5.6)	1303 (6.1)	143 (6.0)
Cesarean section	11,262 (18.8)	4321 (18.2)	474 (18.7)	11,516 (18.5)	3959 (18.6)	485 (20.2)
Missing	97 (0.2)	32 (0.1)	3 (0.1)	98 (0.2)	28 (0.1)	5 (0.2)

*Mother complication during pregnancy*						
Pregnancy induced hypertension, n (%)						
No	58,110 (96.9)	23,031 (96.9)	2450 (96.8)	60,496 (97.0)	20,663 (96.8)	2314 (96.3)
Yes	1873 (3.1)	726 (3.1)	80 (3.2)	1905 (3.1)	679 (3.2)	88 (3.7)
Preexisting hypertension, n (%)						
No	59,096 (98.5)	23,420 (98.6)	2478 (97.9)	61,517 (98.6)	21,007 (98.4)	2348 (97.8)
Yes	887 (1.5)	337 (1.4)	52 (2.1)	884 (1.4)	335 (1.6)	54 (2.2)
Preexisting diabetes mellitus, n (%)						
No	59,305 (98.9)	23,507 (99.0)	2501 (98.9)	61,711 (98.9)	21,115 (98.9)	2364 (98.4)
Yes	678 (1.1)	250 (1.1)	29 (1.2)	690 (1.1)	227 (1.1)	38 (1.6)
Gestational diabetes mellitus, n (%)						
No	58,345 (97.3)	23,108 (97.3)	2468 (97.6)	60,721 (97.3)	20,750 (97.2)	2322 (96.7)
Yes	1638 (2.7)	649 (2.7)	62 (2.5)	1680 (2.7)	592 (2.8)	80 (3.3)
Hypothyroidism, n (%)						
No	59,266 (98.8)	23,472 (98.8)	2506 (99.1)	61,637 (98.8)	21,111 (98.9)	2375 (98.9)
Yes	717 (1.2)	285 (1.2)	24 (1.0)	764 (1.2)	231 (1.1)	27 (1.1)
Anemia medication (iron pill), n (%)						
No	34,408 (57.4)	13,823 (58.2)	1478 (58.4)	35,897 (57.5)	12,308 (57.7)	1423 (59.2)
Yes	25,575 (42.6)	9934 (41.8)	1052 (41.6)	26,504 (42.5)	9034 (42.3)	979 (40.8)
Education, n (%)						
≤high school	20,503 (34.2)	9034 (38.0)	1169 (46.2)	21,108 (33.8)	8648 (40.5)	1224 (51.0)
≥college/university	38,772 (64.6)	14,401 (60.6)	1312 (51.9)	41,049 (65.8)	12,601 (59.0)	1162 (48.4)
Missing	708 (1.2)	322 (1.4)	49 (1.9)	244 (0.4)	93 (0.4)	16 (0.7)
Smoking during pregnancy, n (%)						
Never	35,736 (59.6)	12,707 (53.5)	1168 (46.2)	37,547 (60.2)	11,282 (52.9)	1065 (44.3)
Quit before pregnant	14,094 (23.5)	5589 (23.5)	521 (20.6)	14,761 (23.7)	5023 (23.5)	541 (22.5)
Quit after pregnant	7033 (11.7)	3791 (16.0)	540 (21.3)	7378 (11.8)	3569 (16.7)	504 (21.0)
Current smoking	2246 (3.7)	1247 (5.3)	232 (9.2)	2273 (3.6)	1239 (5.8)	259 (10.8)
Missing	874 (1.5)	423 (1.8)	69 (2.7)	442 (0.7)	229 (1.1)	33 (1.4)
Congenital malformation, n (%)						
No	59,432 (99.1)	23,508 (99.0)	2511 (99.3)	61,849 (99.1)	21,112 (98.9)	2371 (98.7)
Yes	551 (0.9)	249 (1.1)	19 (0.8)	552 (0.9)	230 (1.1)	31 (1.3)

*K6: Kessler Psychological Distress Scale

The mothers who experienced severe distress (K6 ≥ 13) in the early and mid-late pregnancy period were younger, with a higher prevalence of preterm birth and low birth weight of newborns. In addition, they were currently smoking and had lower educational levels.

### Distress during pregnancy and risk of the newborn’s low Apgar score at 5 minutes of life

Table 2 presents the association between maternal distress at different stages of pregnancy and the risk of a low 5-min Apgar score. Maternal distress during mid-late pregnancy was associated with an increased risk of low Apgar score but was not during early pregnancy. Compared to low distress (K6 < 5) during mid-late pregnancy, the multivariable ORs (95%CI) of low Apgar score were 1.22 (1.05–1.42) for moderate distress (K6 score: 5–12) and 1.42 (1.00–2.01) for severe distress (K6 score ≥13) (p for trend = 0.002). The multivariable OR (95% CI) of low Apgar score with moderate-to-severe distress (K6 ≥ 5) was 1.25 (1.08–1.44) compared to low distress. The multivariable OR (95% CI) of low Apgar score per five-point increment in K6 was 1.16 (1.06–1.26).

**Table 2:** Odds ratios of low Apgar score by pregnancy distress levels

	**Distress in early pregnancy period** **(K6 score)**			**OR for 5 points increment in K6**	**p for trend**	**Distress in mid late pregnancy period** **(K6 score)**			**OR for 5 point- increment in K6**	**p for trend**
	
	**0–4**	**5–12**	**≥13**			**0–4**	**5–12**	**≥13**		
Number at risk	59,983	23,757	2,530			62,401	21,342	2,402		
Number of cases (%)	619 (1.0)	278 (1.2)	25 (1.0)			589 (0.9)	251 (1.2)	35 (1.5)		
Crude OR (95%CI)	1.00	1.14 (0.99–1.31)	0.96 (0.64–1.43)	1.06 (0.97–1.15)	0.23	1.00	1.25 (1.08–1.45)	1.55 (1.10–2.19)	1.18 (1.09–1.28)	<0.001
	1.00	1.12 (0.97–1.28)				1.00	1.28 (1.11–1.48)			
Multivariable OR (95%CI)*	1.00	1.11 (0.96–1.28)	0.91 (0.61–1.37)	1.04 (0.95–1.13)	0.43	1.00	1.22 (1.05–1.42)	1.42 (1.00–2.01)	1.16 (1.06–1.26)	0.002
	1.00	1.09 (0.95–1.26)				1.00	1.25 (1.08–1.44)			

*Adjusted for mother’s age, obstetrical factors (parity, and mode of delivery), mother’s complication during pregnancy (pregnancy induced-hypertension, preexisting hypertension, preexisting diabetes mellitus, gestational diabetes mellitus, hypothyroidism and anemia medication), education, smoking during pregnancy, and congenital malformation.

Table 3 shows the adjusted OR (95% CI) of low Apgar score at 5 minutes, stratified by gestational age and birth weight according to distress during early and mid-late pregnancy periods. The risk of low Apgar score was confined to preterm birth (≤36 weeks) and low birth weight (<2500 g) with moderate and severe distress in mid-late pregnancy compared to those with low distress. The OR (95% CI) of moderate and severe distress were 1.44 (1.11–1.87) and 2.02 (1.19–3.44), respectively in preterm birth and 1.03 (0.85–1.24) and 0.95 (0.57–1.57), respectively in term birth (p for interaction = 0.01). The corresponding OR (95% CI) was 1.41 (1.10–1.81) and 1.86 (1.11–3.12) for low birth weight and 1.06 (0.87–1.28) and 1.01 (0.61–1.68) for normal birth weight (p for interaction = 0.02).

**Table 3:** Odds ratios of low Apgar score by distress, stratified by gestational age and birth weight

	**Distress in early pregnancy period** **(K6 score)**			**P for trend**	**Distress in mid-late pregnancy period** **(K6 score)**			**P for trend**
	
	**0–4**	**5–12**	**≥13**		**0–4**	**5–12**	**≥13**	
**Gestational age at birth**								
**≤36 weeks (preterm)**								
Mean (SD)	34.5 (2.6)	34.5 (2.6)	34.5 (2.7)		34.7 (2.2)	34.4 (2.6)	34.2 (2.8)	
Number at risk	2,613	1,074	117		2,521	1,045	134	
Number of cases (%)	227 (8.7)	106 (9.9)	9 (7.7)		174 (6.9)	103 (9.9)	19 (14.2)	
Crude OR (95% CI)	1.00	1.15 (0.90–1.47)	0.88 (0.44–1.75)	0.53	1.00	1.48 (1.14–1.90)	2.23 (1.34–3.71)	<0.001
Multivariable OR (95%CI)*	1.00	1.16 (0.90–1.49)	0.92 (0.44–1.84)	0.50	1.00	1.44 (1.11–1.87)	2.02 (1.19–3.44)	<0.001

**≥37 weeks (term)**								
Mean (SD)	39.0 (1.1)	39.1 (1.1)	39.1 (1.2)		39.1 (1.1)	39.0 (1.1)	39.0 (1.2)	
Number at risk	57,370	22,683	2,413		59,880	20,297	2,268	
Number of cases (%)	392 (0.7)	172 (0.8)	16 (0.7)		415 (0.7)	148 (0.7)	16 (0.7)	
Crude OR (95% CI)	1.00	1.11 (0.93–1.33)	0.97 (0.59–1.60)	0.43	1.00	1.05 (0.87–1.27)	1.02 (0.62–1.68)	0.67
Multivariable OR (95%CI)*	1.00	1.07 (0.89–1.28)	0.92 (0.56–1.53)	0.67	1.00	1.03 (0.85–1.24)	0.95 (0.57–1.57)	0.96

P for interaction		0.88				0.01		

**Birth weight**								
**<2500 g**								
Mean (SD)	2202 (368)	2202 (364)	2229 (345)		2229 (325)	2191 (377)	2164 (382)	
Number at risk	4609	1934	215		4621	1807	226	
Number of cases (%)	242 (5.3)	111 (5.7)	8 (3.7)		191 (4.1)	106 (5.9)	19 (8.4)	
Crude OR (95% CI)	1.00	1.10 (0.87–1.39)	0.70 (0.34–1.43)	0.99	1.00	1.45 (1.13–1.85)	2.13 (1.30–3.48)	<0.001
Multivariable OR (95%CI)*	1.00	1.10 (0.86–1.40)	0.64 (0.31–1.34)	0.89	1.00	1.41 (1.10–1.81)	1.86 (1.11–3.12)	0.001

**≥2500 g**								
Mean (SD)	3100 (334)	3092 (332)	3088 (328)		3100 (334)	3092 (331)	3085 (334)	
Number at risk	55,374	21,823	2315		57,780	19,535	2176	
Number of cases (%)	377 (0.7)	167 (0.8)	17 (0.7)		398 (0.7)	145 (0.7)	16 (0.7)	
Crude OR (95% CI)	1.00	1.13 (0.94–1.35)	1.08 (0.66–1.76)	0.26	1.00	1.08 (0.89–1.31)	1.07 (0.65–1.76)	0.47
Multivariable OR (95%CI)*	1.00	1.10 (0.91–1.32)	1.04 (0.64–1.71)	0.41	1.00	1.06 (0.87–1.28)	1.01 (0.61–1.68)	0.66

P for interaction		0.47				0.02		

*Adjusted for mother’s age, obstetrical factors (parity, and mode of delivery), mother’s complication during pregnancy (pregnancy induced-hypertension, preexisting hypertension, preexisting diabetes mellitus, gestational diabetes mellitus, hypothyroidism and anemia medication), education, smoking during pregnancy and congenital malformation.

## Discussion

Based on the data of 87,765 pairs of mothers and newborns, we found that pregnant women with moderate and severe distress during mid-late pregnancy had a 22% and 42% higher risk of delivering a baby with a low Apgar score at 5 minutes, respectively than women with low distress. A five-point increment in K6 was associated with a 16% higher risk of low Apgar score. Such associations were not observed in the early pregnancy period. Our result was consistent with the findings from a large multiethnic cohort study in the Netherlands [5]. We extended the evidence that such positive associations were confined to preterm births and low birth weight.

The mechanisms by which maternal distress in mid-late pregnancy can affect neonatal health include a direct effect of the enhancing hypothalamic-pituitary-adrenal (HPA) axis to elevate levels of cortisol and stimulate the production of inflammatory cytokines [2] which can affect fetal development by reducing placental blood flow and oxygen delivery to the fetus [12]. An indirect effect is a maternal illness accompanied by poor nutrition, reduced physical activity, and disordered sleep [2], which could raise the risk of hypertension and diabetes, leading to impaired respiratory and cardiovascular function in the fetus [13, 14]. We adjusted maternal hypertension and diabetes in the multivariable model to examine the association between maternal distress and Apgar score, but residual confounding remained, so the indirect effect is not negated.

The strength of our study was a large prospective study with a high response rate under a national birth cohort. In addition, we used the K6 distress measuring tool with sufficient precision for use by the Japanese population [9]. It is important to acknowledge that this study has some limitations as well. First, distress data during pregnancy were obtained from a self-report questionnaire without being diagnosed by psychiatrists. Second, the biomarkers of distress during pregnancy, such as cortisol or catecholamine levels, were not measured, leaving the need for future studies. Third, some conditions, such as general anesthesia during delivery and hospitalization during pregnancy, may affect the results. Fourth, the JECS study is a nationwide cohort study, but variations in socio-cultural factors across different cultures and regions may affect the broader applicability of our findings. Further research in different cultural and regional contexts is needed. Finally, future longitudinal studies with repeated assessments of maternal psychological states and biomarkers or randomized controlled trials to reduce maternal distress are needed.

## Conclusions

In conclusion, pregnant women with moderate to severe distress during mid-late pregnancy but not during early pregnancy were at high risk for giving birth to newborns with low Apgar scores at 5 minutes of life. This association was confined to preterm births and low birth weight.

## Declarations

### Availability of data and material

All data are not available for public use in this study due to ethical and legal restrictions regulated in The Act on The Protection of Personal Information in Japan (Act no. 57 of 30, May 2003, amendment on September 9, 2015). Restrictions on ethical use of data are also limited and regulated in the Ethical Guidelines for Medical and Health Research Involving Human Subjects enforced by the Japan Ministry of Education, Culture, Sports, Science and Technology and the Ministry of Health, Labor and Welfare in Japan. If need the legal use and information, please contact the following email: jecs-en@nies.go.jp For the email that can be contacted, the responsible person in this email is Dr. Shoji F. Nakayama, JECS Program Office, National Institute for Environmental Studies.
